# Phytochemical and Flavor Characteristics of Mulberry Juice Fermented with *Lactiplantibacillus plantarum* BXM2

**DOI:** 10.3390/foods13172648

**Published:** 2024-08-23

**Authors:** Xuefang Guan, Dazhou Zhao, Tian Yu, Shaoquan Liu, Shuying Chen, Junyang Huang, Gongti Lai, Bin Lin, Juqing Huang, Chengchun Lai, Qi Wang

**Affiliations:** 1 Institute of Food Science and Technology, Fujian Academy of Agricultural Sciences, Fuzhou 350003, China; guan-619@163.com (X.G.); a18060772792@163.com (S.C.); laigongti@faas.cn (G.L.); linbin591@163.com (B.L.); jq_huang@zju.edu.cn (J.H.); lccisland@163.com (C.L.); 2 Key Laboratory of Processing of Subtropical Characteristic Fruits, Vegetables and Edible Fungi, Ministry of Agriculture and Rural Affairs of China, Fuzhou 350002, China; 3 Bio-Fermentation Research Center, Xiamen Yuanzhidao Biotechnology Co., Ltd., Xiamen 361028, China; zhao@yzdbio.com (D.Z.); ytcau@163.com (T.Y.); july@yzdbio.com (J.H.); 4 Department of Food Science and Technology, National University of Singapore, Science Drive 2, Singapore 117542, Singapore; fstlsq@nus.edu.sg

**Keywords:** fermented mulberry juice, *Lactiplantibacillus plantarum*, lactic acid bacteria, metabolomics, aroma

## Abstract

Fermentation of mulberry juice not only improves its shelf life, but also effectively enhances their flavor and nutritional quality. This study elucidated the phytochemical and flavor characteristics of mulberry juice fermented with *Lactiplantibacillus plantarum* BXM2, originally isolated from naturally fermented fruit beverage, through widely targeted metabolomics. The fermentation produced the unique flavor of fermented juice and decreased the pH from 4.15 to 3.19. The metabolomic analysis detected 907 non-volatile metabolites, from which 359 significantly different non-volatile metabolites (up 238, down 121) were screened out. Among 731 identified volatile metabolites, 26 flavor substances were the major contributors to the flavor differences between fermented and unfermented mulberry juices. It is hypothesized that lipid metabolism and amino acid catabolism are crucial pathways for the flavor enhancement of mulberry juice fermented with *L. plantarum* BXM2. Meanwhile, significant increases of the contents of a variety of bioactive substances, such as indole-3-lactic acid, octadeca-9,12,15-trienoic acid, di-/tri-peptides, etc., conferred additional health potential to BXM2-fermented mulberry juice.

## 1. Introduction

Mulberry, a plant belonging to the genus *Morus* of the family Moraceae, has been widely cultivated in China, India, Japan and many other countries of the world. Mulberry fruit has long been used as a nutritional agricultural product with medicinal properties since it is known to contain considerable amounts of biologically active ingredients, including phenolic acids, flavonols, amino acids, vitamins, minerals and anthocyanins [1,2], which have been associated with several potential pharmacological properties including protecting eyesight, anti-cholesterol, antioxidative, anti-diabetic and anti-obesity effects [3]. However, due to its high water content (about 80%) and no outer shell protection, it is difficult to preserve mulberry fruits. Therefore, in addition to fresh consumption, most of mulberry fruits are usually processed into a variety of products such as juice, wine, yogurt, jam and dried fruit. To respond to consumer demand for natural, healthy and ready-to-drink products, the production of fruit-based beverage, which contain valuable nutrients that promote health benefits and reduce the risk of diseases, has recently increased. Mulberry beverage has broad market prospects and is a key processing approach to solving the problem of short shelf life of fresh mulberry fruits. However, the existing mulberry beverages exhibit significant homogeneity and suffer from various issues such as the decline in nutritional quality and aroma after sterilization, which affects their nutritional value, flavor quality, and market competitiveness.

Lactic acid bacterial (LAB) fermentation has been used as one of the oldest, most economical and most valuable methods to maintain and/or improve the nutritional, sensory, safety and shelf-life of fruits [4]. Increasing evidences have shown that the fermentation of fruit with LAB can significantly increase the contents of various bioactive substances [5,6]. LAB can utilize various fruit components, i.e., sugar, protein, amino acids, organic acids, polyphenols and other nutrients, through various metabolic pathways, such as proteolysis, amino acid catabolism, lipolysis, fatty acid metabolism, citrate conversion, and phenolic acid degradation [7]. Moreover, probiotic bacteria can produce various volatile flavor compounds during fermentation process, contributing to the improvement of organoleptic characteristics of fermented products [8].

Therefore, the combination of LAB fermentation and mulberry juice can be considered as an effective strategy to not only greatly extend the shelf life of fermented beverages, but also improve its flavor quality and potential health benefits. A recent study indicated that fermentation with three *Lactiplantibacillus* strains (*L. plantarum*, *L. acidophilus* and *L. paracasei*) contributed to a significant increase of total anthocyanin, phenolic, and flavonoid concentrations of fermented mulberry juice, and an improvement of antioxidant activity [9]. Moreover, another study found that *L. plantarum* could produce beneficial metabolites that can ameliorates colorectal tumorigenesis [10].

Widely targeted metabolomics analysis has been extensively used to reveal the mechanism of various processing methods for the quality improvement of processed products [11]. Based on this advanced technology, it is possible to obtain a comprehensive qualitative and quantitative metabolic profile of fermented mulberry beverages by distinct LAB strains. In a previous research, a *Lactiplantibacillus plantarum* (previously known as *Lactobacillus plantarum*) strain BXM2, isolated from naturally fermented honey passion fruit beverage and identified by our research group [12], was well adapted to grow in mulberry juice, and able to significantly enhance the flavor and quality of mulberry beverage. The objective of this present study is to investigate the changes of non-volatile and volatile metabolites of mulberry juice before and after *L. plantarum* BXM2 fermentation through widely targeted metabolomics. The results would provide valuable data and information for the development of unique fermented mulberry juice with improved nutritional value and flavor quality.

## 2. Materials and Methods

### 2.1. Materials and Reagents

*L. plantarum* BXM2 (patent number: ZL201910682492.X) was provided by our laboratory. Black mulberry fruits were harvested at commercial maturity from a commercial mulberry field close to Shantou City, Guangdong Province, China, and immediately transported to the laboratory. Methanol, acetonitrile, and n-hexane were all chromatographic pure and purchased from Merck (Darmstadt, Germany); Formic acid (chromatographic pure) was purchased from Aladdin (Los Angeles, CA, USA), 3-Hexanone-2,2,4,4-d4 (GC-MS standard, chromatographic pure) was purchased from CDN ISOTopes (Quebec, QC, Canada), UPLC standards were purchased from Sigma Aldrich (Saint Louis, MO, USA). Sodium chloride (analytical grade) was obtained from Sinopharm (Shanghai, China). Sucrose was purchased from a local retail supermarket.

### 2.2. Fermentation of Mulberry Juice with BXM2

Cleaned fresh mulberry fruits were mixed with sucrose (9:1, wt/wt) and homogenized using a laboratory blender. The mulberry juice was equally divided, packaged, and sterilized using a steam pressure cooker at 100 °C for 20 min. The samples without fermentation were used as controls (CK), while the rest of samples were used for the fermentation experiment (FM). To prepare the inoculum, *L. plantarum* BXM2 was cultivated in MRS broth at 37 °C for 24 h. Then, a bacterial suspension (approximately 10 log CFU/mL) was added to the sterilized mulberry juice at a ratio of 1:1000 (*v*/*v*) and the mixtures were statically cultured at a constant temperature of 37 °C. To determine the optimum fermentation time, samples were taken at different fermentation times (0 h, 12 h, 24 h, 48 h, 72 h, 96 h) to measure the changes of pH and viable cell counts, and the determination of each sample was conducted in triple. The pH was measured in the laboratory with a Sartorius PB-10 pH meter (Beijing, China). Viable cell counts were determined according to our previous report and expressed as log CFU/mL [12].

### 2.3. Analysis of Organoleptic Characteristics

The organoleptic characteristics of the samples from a CK group (unfermented mulberry juice) and a FM group (BXM2-fermented mulberry juice at 37 °C for 72 h) were assessed via quantitative descriptive analysis (QDA) according to a previous method with minor modifications [13]. The products tested were safe for consumption. The tasting group consisted of 16 well-trained Master students from College of Food Science, Fujian A&F University, China, who possessed professional sensory capacities for identifying and describing food flavor. The panelists were required to evaluate organoleptic characteristics of the samples through orthonasal (sniffing) and retronasal olfaction (sipping and swallow). A 10-point rating scale, where 1 corresponded to ‘extremely unpleasant’ and 10 to ‘extremely pleasant’, was used to perform the panel sensory test including the following attributes: overall, aroma intensity, sourness, fermented, fatty, fruitiness, floral, berry-like.

### 2.4. Analysis of Non-Volatile Metabolites

UPLC-MS/MS detection was performed according to a previously described method [14]. Briefly, the samples from CK and FM groups were pre-frozen in a −80 °C refrigerator overnight, and then lyophilized with a vacuum freeze dryer. Then, 50 mg of freeze-dried sample was added to 1.2 mL of 70% methanol solution, which was pre-heated in a 70 °C water bath. The mixture was vortexed for 15 min, centrifuged at 4 °C and 6000 rpm for 3 min. The collected supernatant was then filtered through a 0.22 μm pore size microfilter membrane. The obtained metabolite extracts were transferred to the injection bottles for metabolomics analysis, with three replicates for each group. Quality control (QC) samples were prepared by mixing extracts and inserting one QC sample for every two samples analyzed.

UPLC-MS/MS-based widely targeted metabolomics analysis included UPLC (ExionLC AD System, Sciex, Framingham, MA, USA) and MS/MS (Applied Biosystems Sciex 4500 QTRAP, Framingham, MA, USA) using an Agilent SB-C18 column (2.1 mm × 100 mm). An elution gradient program was set for chromatographic separation with mobile phase A (pure water with 0.1% formic acid) and mobile phase B (acetonitrile with 0.1% formic acid) as follows: 0 to 9 min, 95 to 5% A and 5 to 95% B; 9 to 10 min, 5% A and 95% B; 10 to 11.1 min, 5 to 95% A and 95 to 5% B; 11.1 to 14 min, 95% A and 5% B. The flow rate was 0.35 mL/min, the column temperature was 40 °C, and the injection volume was 2 μL. The MS parameters were as follows: the electrospray ionization (ESI) source temperature was set at 550 °C; the ion spray voltage (IS) was 5500 V (positive ion mode)/−4500 V (negative ion mode); the gas source I (GSI), gas source II (GSII), and curtain gas (CUR) were set at 50, 60, and 25 psi; the collision-induced dissociation (CID) parameter was set to high. Triple quadrupole (QQQ) scans employed the multiple reaction monitoring (MRM) mode, and the collision nitrogen gas was set to medium. Through the optimization of declustering potential (DP) and collision energy (CE), the DP and CE of each MRM ion pair were obtained. The MRM ion pairs were regularly monitored during each period based on the eluted metabolites.

Based on internal databases and publicly available metabolite databases, such as HMDB, METLIN and MassBank, Analyst (Version 1.6.2) and Multiquant software (Version 3.0.2) packages were used for the analysis of mass spectrometry data. Principal component analysis (PCA), Partial Least Squares-Discriminant Analysis (PLS-DA), and Orthogonal Partial Least Squares (OPLS-DA) models were employed to classify the samples. R^2^X, R^2^Y, and Q^2^ values were used to evaluate the model quality [15,16]. 2-chlorophenylalanine (1 mg/L, J&K Chemical Co., Shanghai, China) was used as an internal standard for the semi-quantitative method, which was applied to quantitatively calculate the components of the samples. OPLS-DA was used to identify significant differences between samples and obtain variable importance in projection (VIP). Single-variable statistical methods were used to calculate the fold change (FC) value and *p*-value of compounds in the comparison group. Under the screening conditions of VIP > 1 and fold change ≥ 2 or ≤0.5, significantly different substances between samples were obtained, and clustering statistical analysis was conducted to analyze the changes in substances with and without BXM2 fermentation.

### 2.5. Analysis of Volatile Metabolites

The extraction and detection method for volatile metabolites was based on the reported method with minor modifications [17]. Briefly, 1.00 g of each sample was transferred into a 20-mL headspace vial, and 5 mL of saturated NaCl solution and 20 μL of internal standard solution (10 μg/mL) were added. An automatic headspace (HS) solid-phase microextraction (SPME) instrument (CTC Analytics AG, SPME Arrow, Zwingen, Switzerland) was used for HS-SPME extraction of the samples. Specifically, to extract the volatile compounds, an aged 120 µm DVB/CWR/PDMS extraction fiber was inserted into the sealed HS vial, incubated at 60 °C for 5 min, followed by a HS extraction for 15 min. Then, the fiber was desorbed for 5 min at 250 °C. Subsequently, the obtained sample extracts were analyzed using a GC-MS (Agilent, 8890-7000D, Santa Clara, CA, USA) instrument. The chromatographic conditions were set as follows: DB-5MS capillary column (30 m × 0.25 mm × 0.25 μm, Agilent J&W Scientific, Folsom, CA, USA), carrier gas: high-purity helium (He) with a flow rate of 1.2 mL/min, injection port temperature: 250 °C. The temperature program was as follows: 40 °C for 3.5 min, ramp to 100 °C at 10 °C/min, ramp to 180 °C at 7 °C/min, ramp to 280 °C at 25 °C/min, and hold for 5 min. The mass spectrometry conditions were set as follows: electron energy: 70 eV, transfer line temperature: 280 °C, ion source temperature: 230 °C, and scan mode: selected ion monitoring (SIM) for qualitative and quantitative ion precise scanning (GB 23200.8-2016) [18].

Based on the internal database, MassHunter software (Version B.07.00) was used for qualitative and quantitative analysis of the original mass spectrometry data to obtain the types and peak areas of volatile substances in the samples. 3-Hexanone-2,2,4,4-d4 (10 μg/mL)was used as an internal standard for the semi-quantification of the volatiles compounds by comparison with the peak areas of the internal standard. PCA was employed to assess the differences among the samples, and OPLS-DA was used to identify significant differences among the samples. VIP, FC, and *p*-values were applied to screen for different metabolites. Cluster statistical analysis was conducted to investigate the dynamic changes of different volatiles. The relative odor activity value (ROAV) of volatile was calculated to assess the contribution of compounds to the aroma formation of BXM2-fermented mulberry beverages. The descriptions of the aroma of the substances were sourced from multiple websites such as http://www.thegoodscentscompany.com (accessed on 11 January 2024), http://perflavory.com (accessed on 11 January 2024), http://www.odour.org.uk/odour/index.html (accessed on 12 January 2024), and http://foodflavorlab.cn/#/home (accessed on 12 January 2024). The threshold values were referenced from the literature [19,20].

### 2.6. Statistical Analysis

PCA was conducted using the R software (version 3.5.1). OPLS-DA was performed by MetaboAnalystR of R (Version 1.0.1). Heatmaps and associated hierarchical clustering were performed in R using the package ComplexHeatmap (2.8.0). IBM SPSS Statistics 25 software was used to perform one-way analysis of variance (ANOVA) and Duncan’s test on experimental data, and *p* < 0.05 was considered as statistically significant.

## 3. Results and Discussion

### 3.1. Growth of L. plantarum BXM2 and pH Changes during Fermentation

**Figure 1:**
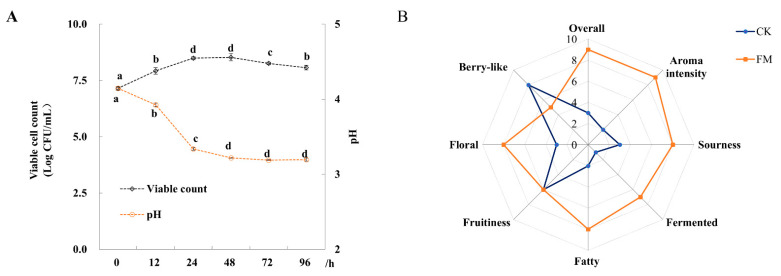
Changes of pH, viable cell count and organoleptic characteristics of mulberry juice samples (*n* = 3). (**A**) Changes of pH and viable cell count during 96 h of fermentation; (**B**) Radar plot of CK (unfermented juice) and FM (fermented juice) samples based on the organoleptic characterizations. Different letters represent significant differences (*p* < 0.05).

The changes of viable cell counts and pH value of mulberry juice fermented with *L. plantarum* BXM2 during 96 h of fermentation are shown in Figure 1A. The initial viable bacterial count in the mulberry juice was 7.15 ± 0.08 log CFU/mL. After 48 h of fermentation, the viable count reached the peak value of 8.53 ± 0.16 log CFU/mL, which then decreased slowly to 8.07 ± 0.10 log CFU/mL at the end of the fermentation (96 h). During the fermentation of mulberry juice, the initial pH value of 4.14 ± 0.03 rapidly decreased to 3.22 ± 0.01 after 48 h and then remained nearly constant until the end of fermentation.

### 3.2. Evaluation of Organoleptic Characteristics

Before the analysis of volatile compounds, QDA was used to evaluate the changes of organoleptic characteristics of the fermented mulberry juice relative to the unfermented juice. It was found that 72 h of fermentation with *L. plantarum* BXM2 resulted in the best sensory characteristics. Moreover, QDA was applied to compare the aromatic characteristics between the sample with 72 h of fermentation and unfermented sample (Figure 1B). Compared to unfermented mulberry juice, fermentation with strain BXM2 decreased the berry flavor, but significantly intensified the sour, fermented, floral and fatty flavors, and thus markedly enhancing the aroma intensity and overall quality of fermented mulberry juice. The results indicated that strain BXM2 would be suitable for mulberry fermentation, and changes in volatile and non-volatile metabolites should be further explored.

### 3.3. Changes of Non-Volatile Metabolites

#### 3.3.1. Metabolite Composition and OPLS-DA Analysis

**Figure 2:**
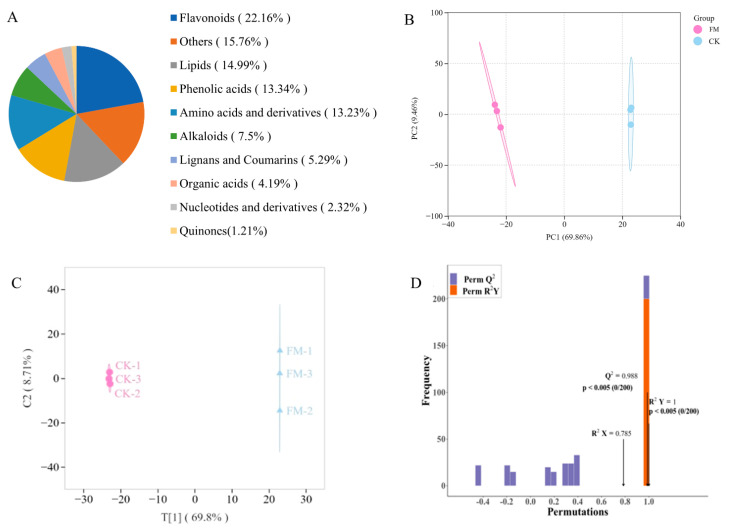
Non-volatile compound composition and multivariate analysis. (**A**) Non-volatile metabolite category composition; (**B**) Principal component analysis; (**C**) OPLS-DA scores plot; (**D**) Validation of OPLS-DA model by permutation testing.

A widely targeted metabolomics analysis was conducted on metabolites using UPLC-MS/MS, and a total of 907 metabolites were detected in all samples (Figure 2A), including 68 alkaloids, 127 amino acids and derivatives, 201 flavonoids, 48 lignans and coumarins, 136 lipids, 21 nucleotides and derivatives, 38 organic acids, 121 phenolic acids, 11 quinones, and 143 others. The OPLS-DA model was employed to preliminarily identify significant differences between samples. As shown in Figure 2B,C, there was a significant difference between CK and FM groups, indicating that varying contents of non-volatile metabolites resulting from strain BXM2 fermentation. Model validation (Figure 2D) revealed that the OPLS-DA model was successfully constructed with R^2^X, R^2^Y, and Q^2^ values of 0.785, 1.00, and 0.988, respectively, indicating that the model had good interpretability and credibility. Therefore, it is suitable for exploring the differential non-volatile metabolites.

#### 3.3.2. Statistics of Differential Non-Volatile Metabolites

**Figure 3:**
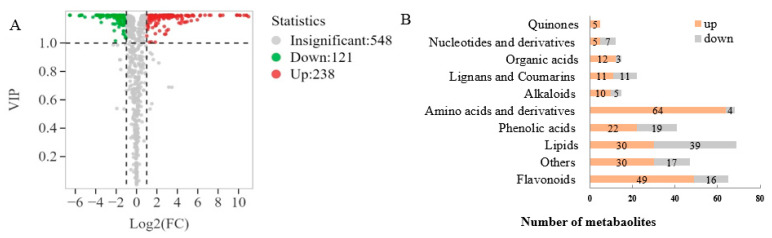
Screening and analysis of differential non-volatile metabolites. (**A**) Volcano plots of differential metabolites; (**B**) Bar chart of the number of the differential metabolites.

**Figure 4:**
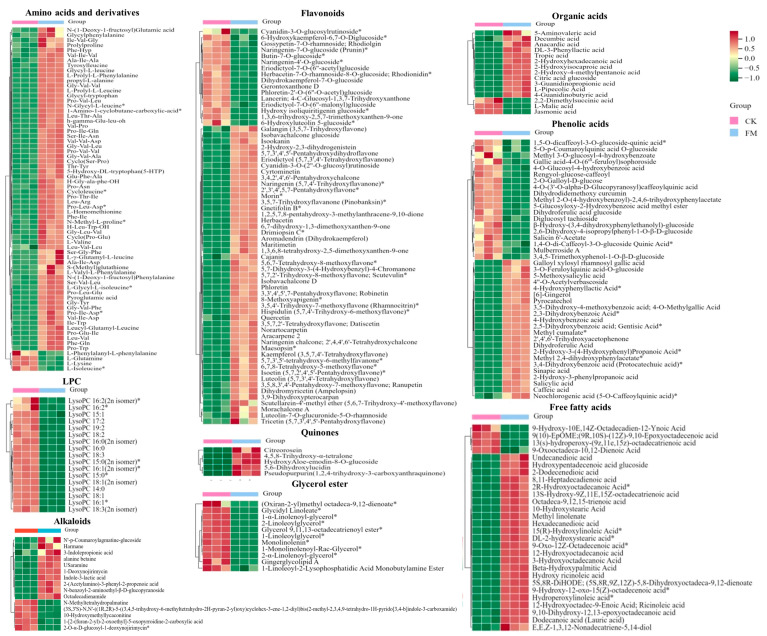
Heatmap of the levels of main differential non-volatile metabolites between CK and FM groups. CK, unfermented mulberry juice; FM, fermented mulberry juice. “*” indicates the presence of isomers of the substance.

Based on the VIP values obtained from the OPLS-DA model (Figure 3A), using VIP > 1 and fold change ≥ 2 or ≤0.5 as the selection criteria, a total of 359 differential metabolites were obtained, including 238 upregulated and 121 downregulated metabolites. Further statistical analysis (Figure 3B) revealed that amino acids and derivatives (up 64, down 4), flavonoids (up 49, down 16), organic acids (up 12, down 3), quinones (up 5), alkaloids (up 10, down 5) and phenolic acids (up 22, down 19) were the upregulated subcategories, while nucleotides and derivatives (up 5, down 7) were the downregulated category. Upon examination of the secondary classification (Figure 4), it was found that free fatty acids (up 24, down 4) exhibited an upregulated trend, while lysophosphatidylcholine (LPC, down 17) and glycerol ester (down 11) were downregulated.

#### 3.3.3. Changes of Non-Volatile Metabolites after *L. plantarum* BXM2 Fermentation

To gain a more specific understanding of the differences in non-volatile metabolites, clustering heatmap analysis (Figure 4) was conducted on significantly different amino acids and derivatives, flavonoids, phenolic acids, organic acids, alkaloids and lipids. The full set of data is shown in Table S1.

After strain BXM2 fermentation, 64 upregulated and 4 downregulated amino acids and derivatives were found, indicating that the fermentation could dramatically increase amino acids and derivatives. Amino acids contribute to distinct taste properties such as sweet, sour, bitter, and umami [21]. For instance, alanine (Ala), glutamine (Gln), proline (Pro), serine (Ser), and threonine (Thr) contribute to sweet tasting, glutamic-acid (Glu) and aspartic acid (Asp) are responsible for umami/sour tasting, while bitter tasting amino acids include leucine (Leu), isoleucine (Ile) and valine (Val) [22]. Moreover, some small peptides, such as Pro-containing peptides, Glu-containing peptides and Val-containing peptides, could also exhibit sweet, umami and bitter tastes [23]. Therefore, these significantly increased amino acids and small peptides might provide the complex taste of sweet, umami and moderate bitterness of fermented mulberry juice. Four down-regulated amino acids and derivatives, including L-glutamine, L-isoleucine, L-lysine, L-phenylalanyl-L-phenylalanine, may be related to the growth of the bacteria or the formation of various flavor substances. *Lactobacillus* can produce proteolytic enzymes causing the degradation of proteins to bioactive peptides and amino acids during the fermentation [24]. Some small peptides (di-peptides and tri-peptides) not only exhibit various health benefits, including antioxidant, antibacterial, anti-inflammatory, antithrombotic and blood pressure lowering [7], but also improve the environmental tolerance of fermentation microorganisms and promote fermentation efficiency [25]. It is also notable that strain BXM2 fermentation significantly increased the content of 5-hydroxy-DL-tryptophan (5-HTP), which is a precursor to the neurotransmitter serotonin with anti-depressant, analgesic and appetite-suppressant activities [26]. Therefore, these upregulated amino acids and derivatives could not only improve the taste and flavor quality, but also enhance the health functions of fermented mulberry juice.

Flavonoids possess various biological activities, including antioxidant, anti-inflammatory, antibacterial, antitumor, and cardiovascular protective functions [27]. After fermentation, 49 flavonoids were significantly upregulated, while 16 were significantly downregulated, indicating that strain BXM2 could increase the flavonoid contents in the fermented mulberry juice. It was reported that LAB are able to secrete various enzymes to hydrolyze complex phytochemicals into simpler forms. In this study, strain BXM2 might hydrolyze glycosylated flavonoids (e.g., cyanidin-3-*O*-glucosylrutinoside, butin-7-*O*-glucoside, naringenin-7-*O*-glucoside) into various simpler forms through its glucosidase activity [28]. Notably, fermentation with strain BXM2 led to significant increases of various flavonoids with biological activity potential, including galangin, isookanin, naringenin, morin, quercitrin, herbacetin, etc., which provides a good foundation for the application of fermented mulberry juice as a functional beverage ingredient. Another study also found that fermentation with lactic acid bacteria significantly increased the content of total phenols and flavonoids, and improve the free radical scavenging ability of fermented mulberry [9]. Meanwhile, the increase of flavonoids can not only enhance the nutritional value, but also play an important role in determining the color, flavor, and mouthfeel of the mulberry beverage and thus directly affecting its quality [29].

*L. plantarum* BXM2 could produce a sour taste and improve the flavor of fermentation product, which is related to the accumulation of organic acids. In this study, 12 kinds of organic acids increased significantly after fermentation, and they were ranked from high to low according to the Log2 FC value: 3-hydroxy-2-phenylpropanoic acid (with a gentle sour taste and aroma), DL-3-phenyllactic acid (with the aroma of benzene ring and sour taste of lactic acid), 2-hydroxy-4-methylpentanoic acid (with typical sour taste), 2-hydroxyisocaproic acid, 4-guanidinobutyric acid, etc. Meanwhile, 3 kinds of organic acids decreased significantly, which were L-malic acid, jasmonic acid, and 2,2-dimethylsuccinic acid. Organic acids with suitable contents are important basic substances for improving the flavor of mulberry juice, providing a refreshing, gentle, and special aroma.

**Figure 5:**
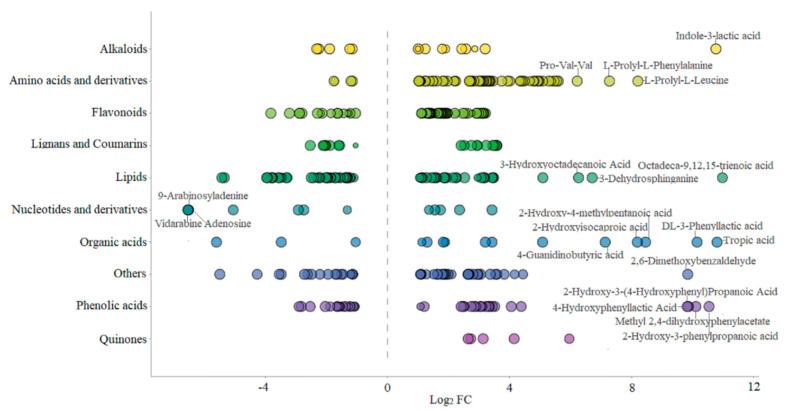
Class scatter plot of differential metabolites (Note: Each point in the figure represents a metabolite, and different colors indicate different classifications. The size of the dot represents the VIP value. The top 10 compounds with the highest Log2 FC are as follows: octadeca-9,12,15-trienoic acid, tropic acid, indole-3-lactic acid, DL-3-phenyllactic acid, 2-hydroxy-3-phenylpropanoic acid, methyl 2,4-dihydroxyphenylacetate, 2-hydroxy-3-(4-hydroxyphenyl)propanoic acid, 2,6-dimethoxybenzaldehyde, 4-hydroxyphenyllactic acid, 2-hydroxy-4-methylpentanoic acid, and L-prolyl-L-leucine).

Lipid metabolites, including free fatty acids (FFA), glycerol ester and lysophosphatidylcholine (LPC) were examined. After fermentation with *L. plantarum* BXM2, 56 of lipid metabolites were elevated, among them 11 glycerol esters, 17 LPCs and 4 FFAs were downregulated, while 24 FFAs were upregulated. Our results suggested that LAB BXM2 fermentation could release various enzymes to promote the conversion of glycerol esters and LPCs into FFAs, which may serve as important precursors of flavor substances [30]. FFAs might be oxidized to produce different hydroperoxides and then decomposed through many different pathways to produce volatile compounds, thereby providing the fermented mulberry juice with a characteristic aromatic flavor [31]. Notably, the lipid compound with the highest Log2 FC (10.98) value was octadeca-9,12,15-trienoic acid (linolenic acid) (Figure 5), which is an essential unsaturated fatty acid for the human body.

Bioactive alkaloids of plant origin play a significant role in human health and medicine. Fermentation with LAB BXM2 increased alkaloids contents (up 10, down 5). Notably, among upregulated alkaloids, there were some alkaloids that had been proven to be strongly related with human health. Indole-3-lactic acid (ILA) and 3-indolepropionic acid, two tryptophan -related metabolites, affect various physiological processes and may contribute to intestinal and systemic homeostasis in health and disease [32]. A recent research by Zhang et al. [10] found that ILA is able to ameliorate the occurrence and progression of colorectal cancer through multiple pathways. 1-Deoxynojirimycin (DNJ), as a potent α-glycosidase inhibitor, has been applied for therapeutic purposes, such as diabetes, HIV and Gaucher’s disease [33].

Among the 41 different phenolic acids (up 22, down 19) examined herein, 4 phenolic acids exhibited Log2 FC values greater than 9.8, namely 2-hydroxy-3-(4-hydroxyphenyl) propanoic acid, 2-hydroxy-3-phenylpropanoic acid, methyl 2,4-dihydroxyphenylacetate, and 4-hydroxybenzoic acid. Phenolic acids may endow foods with unique aromas and flavors if present at sufficient levels, and can also react with alcohols to produce ester compounds to create distinct flavor. Furthermore, tryptophan-derived ILA exhibited a high Log2 FC value of 10.77 (Figure 5), indicating that *L. plantarum* BXM2 can produce a high yield of ILA through the fermentation of mulberry.

In summary, after fermentation with *L. plantarum* BXM2, the contents of amino acids and their derivatives, organic acids, free fatty acids and flavonoids changed significantly, which may be responsible for the improved flavor of fermented mulberry beverage. Additionally, the significant increase of bioactive substances, i.e., dipeptides, tripeptides, linolenic acid, 5-HTP, ILA, and flavonoids can boost the health potential of mulberry beverage.

### 3.4. Changes of Volatile Compounds

#### 3.4.1. Statistical Analysis of Volatile Compounds

**Figure 6:**
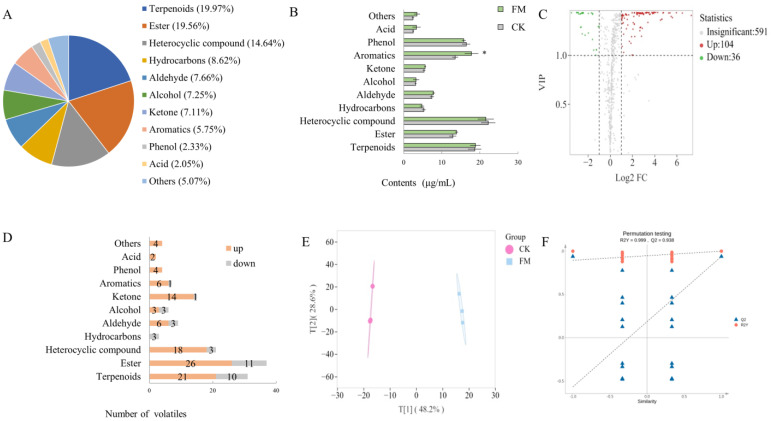
Screening and statistical analysis of differential volatile compounds. (**A**) Pie chart of volatile category composition; (**B**) Bar chart of the contents of volatiles; (**C**) Volcano plots of differential volatiles; (**D**) Bar chart of the number of the differential volatiles; (**E**) OPLS-DA score plot; (**F**) Validation of OPLS-DA model by permutation testing. The * means a significant difference (*p* < 0.05).

HS-SPME-GC-MS was used to detect and analyze the distribution of overall volatile compounds in mulberry juice before and after fermentation with *L. plantarum* BXM2 (Figure 6A). In this study, 731 volatiles were detected, including 146 terpenoids (19.97%), 143 esters (19.56%), 107 heterocyclic compounds (14.64%), 63 hydrocarbons (8.62%), 56 aldehydes (7.66%), 53 alcohols (7.25%), 53 ketones (7.11%), 42 aromatics (5.75%), 17 phenols (2.33%), 15 acids (2.05%), and 37 others (5.07%). As shown in Figure 6B, the volatile categories with higher contents after the fermentation were terpenoids, aromatics, phenols, and esters, with a range of 12.87 to 22.87 μg/mL. Among them, the content of aromatics significantly increased from 13.49 μg/mL to 17.79 μg/mL (*p* < 0.05). To screen for flavor substances that exhibited significant differences before and after fermentation, intergroup volatile difference statistical analysis was performed (Figure 6C) on the 731 detected volatile compounds (screening conditions: VIP > 1, FC ≥ 2 or ≤0.5), and 140 significantly different metabolites were obtained (up 104, down 36). Further analysis of the changes in various categories of volatiles (Figure 6D) revealed that, except for hydrocarbons, the number of upregulated volatiles in other categories was higher than that of downregulated volatiles. It was postulated that terpenoids, esters, heterocyclic compounds, aromatics, phenols, and ketones were the main contributors to the improved flavor of the fermented mulberry juice. The OPLS-DA model analysis (Figure 6E) showed that the CK group clustered in the first and fourth quadrants, while the FM clustered in the second and third quadrants, indicating that the mulberry juice before and after fermentation could be well distinguished based on volatile compounds. OPLS-DA permutation analysis (Figure 6F) indicated that the OPLS-DA model was successfully constructed with high goodness of fit and predictive ability.

#### 3.4.2. Analysis of ROAV of Differential Volatiles

The contribution of flavor substance to the overall flavor of food depends not only on its content but also on its relative odor activity value (ROAV). Volatile compounds with ROAV ≥ 1 are considered the key flavor components in the sample, while those with ROAV between 0.1 and 1 have an auxiliary function in the overall flavor [34]. This study summarized the differential volatiles with ROAV ≥ 0.1 after mulberry fermentation (Table 1). It was found that after fermentation with *L. plantarum* BXM2, there were 26 flavor substances with ROAV greater than 0.1 in the samples (20 up and 6 down). Among the upregulated substances with ROAV ≥ 1, the ester category included decanoic acid, methyl ester (oily, wine, fruity, floral), dodecanoic acid, methyl ester (waxy, soapy, creamy, coconut, mushroom), and methyl salicylate (caramel, pepperminty). These volatiles are important contributors to the lipid and fruity aroma of fermented mulberry juice. Esters can be formed by esterification reactions between free fatty acids and alcohols formed by lipid degradation [35].

The formation of benzene derivatives and naphthalene-like polycyclic aromatic hydrocarbons mainly originates from the degradation of amino acids and the cleavage of fatty acid alkoxy radicals [36]. The total content of aromatic hydrocarbons in the mulberry juice significantly increased after LAB BXM2 fermentation. Among them, 2-methoxy-4-vinylphenol (spicy, raisin), benzene, 1-ethyl-2-methyl- (aromatic), and naphthalene, 1,2-dihydro-1,1,6-trimethyl- (licorice) showed significant increases (ROAV ≥ 1), which were important contributors to the aromatic flavor characteristics of fermented mulberry juice. In the metabolomic analysis, the content of phenylalanine decreased significantly, suggesting that the degradation of phenylalanine might play an important role in the formation of aromatic compounds. Similar result has also been observed in *L. plantarum*-fermented pomegranate juice [37].

Eugenol, the main significantly increased volatile in the volatile phenol category, possesses floral and clove flavors, as well as a vast range of therapeutic properties, including anti-inflammatory, antioxidant, and anticancer properties [38]. The mechanism underlying the formation of eugenol remains unclear, and further research is needed. Three ketones, including 1-(4-methylphenyl)-ethanone, 1-octen-3-one and 2-undecanone were increased in the fermented samples. The incomplete β-oxidation of free fatty acids and amino acid catabolism could lead to the increase of ketones during LAB BXM2 fermentation, which can impart fruity, rose, floral and tea odors [39]. Nonanoic acid was the only acidic compound with ROAV ≥ 1 after LAB BXM2 fermentation, and its odor is waxy, dirty, and cheesy with a cultured dairy nuance [40]. Given that nonanoic acid is a saturated fatty acid containing nine carbon atoms, it is possible that its production may be related to the increase in free fatty acids.

Benzaldehyde and octanal significantly increased in the aldehydes category. Benzaldehyde possesses a characteristic pleasant almond-like odor, and has been widely used as an important fragrant substance in the food, beverage, and cosmetics industries. The biosynthesis of benzaldehyde may originate from the deamination of phenylalanine by the phenylalanine ammonia lyase [41]. Octanal, with its lemon, citrus, and green grass aroma, contains 8 straight-chain carbon atoms and may be derived from the oxidation of unsaturated fatty acids [42].

Alcohols are important components of flavor. In this study, 4-phenyl-2-butanol (with floral, peony, foliage, sweet, mimosa, and heliotrope notes) and 3-mercapto-3-methyl-1-butanol (with roasted, spicy, sweet, and vegetable notes) increased significantly after fermentation, making significant contributions to the formation of floral and fruity flavor. Linear alcohols are produced by the oxidative decomposition of unsaturated fatty acids, while branched alcohols are derived from the degradation of the corresponding branched aldehydes [43].

In summary, after fermentation with *L.plantarum* BXM2, the total content of aromatic compounds in mulberry juice significantly increased, along with notable increases in compounds such as butanoic acid, butyl ester, decanoic acid, methyl ester, methyl salicylate, 2-methoxy-4-vinylphenol, benzene, 1-ethyl-2-methyl-, naphthalene, 1,2-dihydro-1,1,6-trimethyl-, eugenol, 1-octen-3-one, benzaldehyde, octanal, 3-mercapto-3-methylbutanol, and 4-phenyl-2-butanol. These volatiles are the primary contributors to the significant improvement in the flavor characteristics of fermented mulberry juice. Further analysis revealed that the production of methyl laurate, methyl caprate, 1-octen-3-one, pentanoic acid, and octanal may be associated with the degradation of glycerides, LPC, and the formation of FFA. Additionally, the generation of key flavor-enhancing substances such as 2-methoxy-4-vinylphenol, 1-ethyl-2-methyl-benzene, benzaldehyde, and 4-phenyl-2-butanol may be linked to the degradation of phenylalanine. Our results suggest that these volatiles can be produced from different metabolic pathways, e.g., lipid metabolism and amino acid catabolism, which are the main mechanisms for *L. plantarum* BXM2 to create a sustained, rich, and intense aroma during the fermentation.

## 4. Conclusions

*Lactiplantibacillus plantarum* BXM2 is a potential probiotic lactic acid bacterium originally isolated from naturally fermented honey passion fruit beverage. This study analyzed the impact of *L.plantarum* BXM2 fermentation on the nutritional value and flavor quality of mulberry juice. During fermentation, the pH value of mulberry juice decreased from 4.15 to 3.19. The viable cell count reached a peak at 48 h. The fermentation imparted mulberry juice with a sustained, rich, and intense lipid, floral, fruity, fermented, and sour aroma, significantly improving the flavor characteristics of the original mulberry juice. Targeted metabolomics revealed that *L. plantarum* BXM2 had the ability to enhance the levels of amino acids and their derivatives, organic acids, free fatty acids, alkaloids and flavonoids in fermented mulberry juice. The significant increases of indole-3-lactic acid, octadeca-9,12,15-trienoic acid, di-/tri-peptides, etc, might confer additional health potential to BXM2-fermented mulberry juice. The volatile metabolomics indicated that fermentation with LAB BXM2 significantly increased the content of aroma compounds in mulberry juice. These compounds conferred fermented mulberry juice with sustained, intense lipid, floral, fruity, fermented, and sour aroma characteristics. In future studies, the potential health benefits of LAB BXM2-fermented mulberry juice need to be further evaluated in cell models or animal models.

## Figures and Tables

**Table 1:** Relative odor activity values (ROAV) of volatile components in CK and FM groups.

Compounds	Concentration (μg/L)		ROAV		Type	Fold Change
	CK	FM	CK	FM		
**Ester**						
Butanoic acid, 2-methylpropyl ester	1.05 ± 0.03	5.78 ± 0.79	0.11	0.62	up	6.12
Butanoic acid, butyl ester	1.73 ± 0.07	23.45 ± 8.70	0.06	0.84	up	14.93
Decanoic acid, methyl ester	31.19 ± 10.17	81.39 ± 11.28	7.25	18.93	up	2.89
Dodecanoic acid, methyl ester	1.72 ± 0.07	29.75 ± 0.77	0.49	8.50	up	19.31
Methyl salicylate	9.50 ± 0.86	235.66 ± 14.30	0.24	5.89	up	27.61
3-Hexen-1-ol, acetate, (Z)-	61.48 ± 8.18	9.51 ± 0.53	1.98	0.31	down	0.17
Butanoic acid, ethyl ester	17.53 ± 4.53	6.26 ± 4.22	330.70	118.12	down	0.41
Hexanoic acid, ethyl ester	6.41 ± 0.61	1.44 ± 1.39	1.28	0.29	down	0.25
**Aromatics**						
2-Methoxy-4-vinylphenol	15.65 ± 10.31	348.30 ± 33.37	5.22	116.10	up	25.04
Benzene, 1-ethyl-2-methyl-	234.06 ± 25.46	1560.69 ± 226.54	0.65	4.34	up	7.39
Naphthalene, 1,2-dihydro-1,1,6-trimethyl-	2.94 ± 0.30	23.59 ± 0.53	1.18	9.43	up	8.92
**Phenol**						
Eugenol	5.78 ± 3.71	118.61 ± 2.68	2.31	47.44	up	22.91
Phenol, 4-ethyl-2-methoxy-	1.15 ± 0.08	4.06 ± 0.28	0.03	0.12	up	3.91
**Ketone**						
1-(4-methylphenyl)-Ethanone	2.97 ± 0.41	5.52 ± 0.19	0.14	0.26	up	2.06
1-Octen-3-one	9.68 ± 6.38	40.11 ± 6.21	193.61	802.14	up	4.60
2-Undecanone	1.09 ± 0.04	9.23 ± 1.08	0.18	1.49	up	9.45
**Acid**						
Nonanoic Acid	9.21 ± 5.97	61.08 ± 8.41	5.76	38.17	up	7.37
**Aldehyde**						
BenzAldehyde	153.46 ± 11.51	1055.10 ± 147.30	0.44	3.01	up	7.62
Octanal	1.23 ± 0.05	5.75 ± 0.48	1.76	8.22	up	5.19
3-Hexenal, (Z)-; (Z)-	127.89 ± 1.41	14.22 ± 0.79	31.97	3.56	down	0.12
**Alcohol**						
3-Mercapto-3-methylbutanol	3.65 ± 0.27	6.98 ± 1.12	0.91	1.74	up	2.12
4-Phenyl-2-butanol	0.81 ± 0.03	5.58 ± 0.98	0.19	1.30	up	7.73
**Terpenoids**						
4-(1-methylethyl)-Benzaldehyde	6.90 ± 0.75	42.69 ± 6.17	0.02	0.11	up	6.87
**Heterocyclic compound**						
3,6-Dimethyl-2,3,3a,4,5,7a-hexahydrobenzofuran	3.73 ± 0.21	9.39 ± 0.96	0.12	0.31	up	2.81
2-Furanmethanethiol, 5-methyl-	11.99 ± 2.95	1.72 ± 1.66	239.84	34.41	down	0.16
Furan, 2-pentyl-	107.19 ± 28.53	45.28 ± 5.54	17.87	7.55	down	0.47

## Data Availability

The original contributions presented in the study are included in the article and Supplementary Materials, further inquiries can be directed to the corresponding author.

## Supplementary Materials

The following supporting information can be downloaded at: https://www.mdpi.com/article/10.3390/foods13172648/s1, Table S1: The contents of differential non-volatile metabolites between CK and FM groups.
